# Surveillance of International Travel of COVID‐19 Cases (SuITCases) in England

**DOI:** 10.1111/irv.70141

**Published:** 2025-07-30

**Authors:** Mary A. Sinnathamby, Katherine Twohig, Nurin Abdul Aziz, Florence Halford, Asad Zaidi, Katie Harman, Simon Thelwall, Alex Allen, Gavin Dabrera

**Affiliations:** ^1^ COVID‐19 Vaccines and Epidemiology Division UK Health Security Agency London UK

**Keywords:** COVID‐19, pandemic, SARS‐CoV‐2, surveillance, travel

## Abstract

**Introduction:**

The emergence of SARS‐CoV‐2 variants necessitated identification of travel‐associated COVID‐19 cases in England.

**Methods:**

We implemented a novel integrated COVID‐19‐episode‐level travel surveillance system, Surveillance of International COVID‐19 Cases (SuITCases), to assign imported, sporadic or unknown travel status to COVID‐19 cases, using data linkage between two enhanced and two routine data sources.

**Results:**

SuITCases identified 517,988 travel‐associated SARS‐CoV‐2 episodes (3.0% of total), where the two enhanced systems assigned most travel statuses.

**Conclusions:**

Our unique system facilitated rapid identification of travel‐associated COVID‐19 cases, reducing transmission and informing public health actions. Enhanced surveillance data sources should be considered as potential tools for future outbreak investigations and pandemic preparedness.

## 1 Background

Since its emergence in late 2019, the severe acute respiratory syndrome coronavirus 2 (SARS‐CoV‐2) virus has been detected globally with the emergence of several dominant variants of concern [1]. International travel information played a vital role in the early detection and mitigation of SARS‐CoV‐2 transmission to inform public health action [2, 3, 4, 5].

After the first confirmed cases of SARS‐CoV‐2 in Europe were detected in Italy and the United Kingdom at the end of January 2020, many countries experienced importations and rapidly implemented different testing strategies in response such as targeted and passive surveillance systems [6, 7, 8, 9, 10]. For example, Denmark detected its first case associated with travel to Northern Italy in late February 2020, which led to the upscaling of polymerase chain reaction (PCR) testing among suspected cases and linkage to registries for enhanced epidemiological data including travel [5].

Public Health England (now UK Health Security Agency [UKHSA]) collected travel information on the first hundred SARS‐CoV‐2 cases and their contacts using the World Health Organization (WHO)'s First Few X enhanced surveillance protocol [4]. This identified that 51.4% of the initial UK SARS‐CoV‐2 cases up to April 2020 were imported [4].

Internationally identified SARS‐CoV‐2 variants were introduced into the United Kingdom in late 2020 and reinforced the need to categorise travel‐associated/imported SARS‐CoV‐2 cases to understand and mitigate their transmission dynamics.

As such, we describe the development and implementation of an integrated travel surveillance system and provide examples to assess and assign travel exposures amongst COVID‐19 cases including variant cases in England between 01 December 2020 and 30 April 2022.

## 2 Methods

Laboratory‐confirmed SARS‐CoV‐2 cases in England are routinely collected through UKHSA's laboratory‐based Second Generation Surveillance System (SGSS), including patient identifiable demographic and epidemiological data [11, 12]. Cases were linked to validated whole genome sequencing (WGS) results, including variant assignment, processed from the Cloud Infrastructure for Big Data Microbial Bioinformatics (CLIMB) database [12, 13].

### 2.1 Creation of the Integrated Travel Dataset (Surveillance of International Travel of COVID‐19 Cases [SuITCases])

**FIGURE 1:**
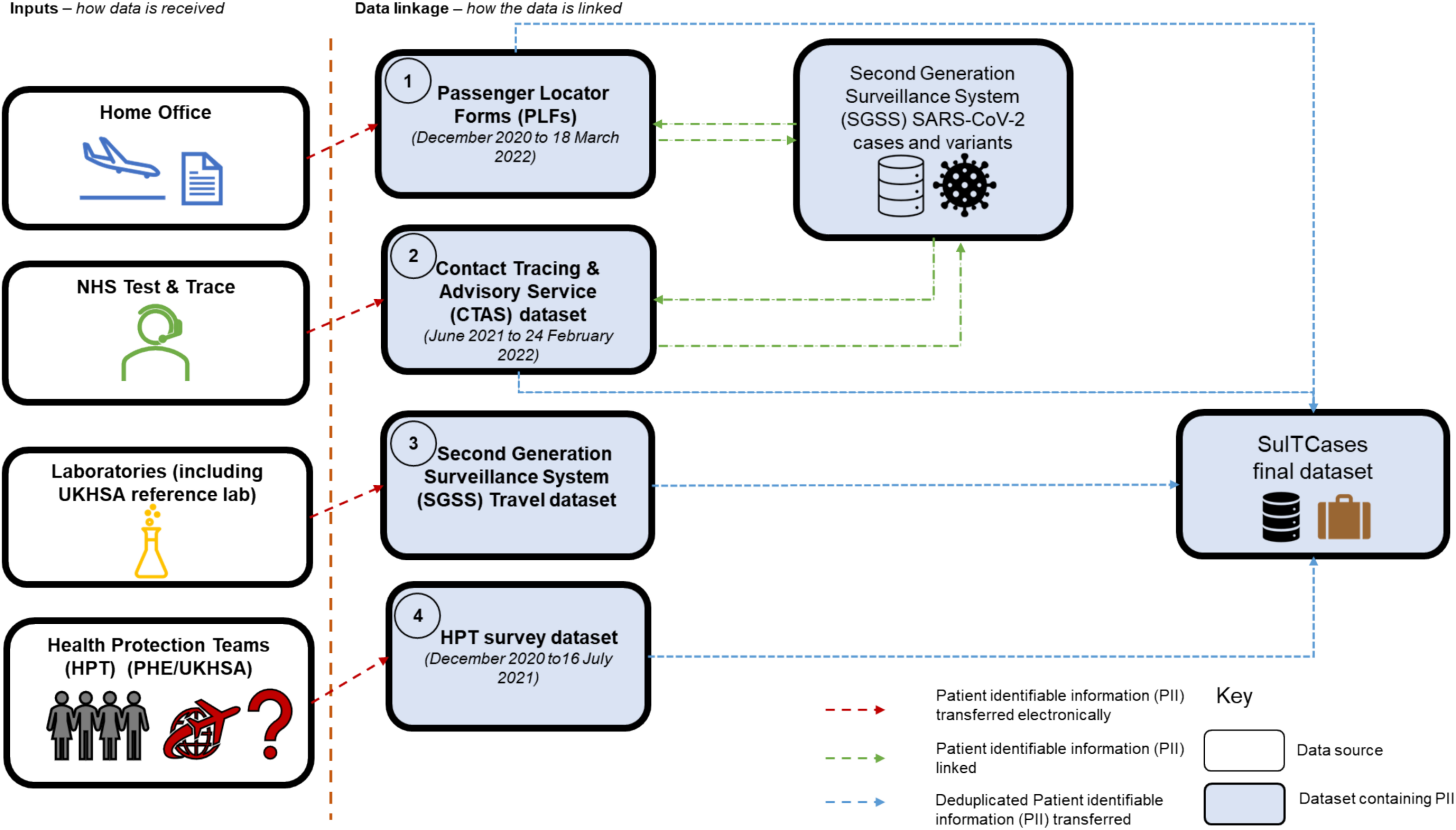
Dataflows for SuITCases.

As the COVID‐19 pandemic progressed, new data collection mechanisms were developed to improve ascertainment of travel information. Datasets required sufficient variables with identifiable information to link to SARS‐CoV‐2 cases. From December 2020 to April 2022, four sources were identified. These included a combination of two modified routine public health surveillance tools and two novel enhanced systems (Figure 1). Enhanced systems are ones which were novel and initiated during the COVID‐19 pandemic. The combination of these sources comprises the integrated travel dataset, SuITCases.

#### 2.1.1 Dataset 1 (Enhanced): Passenger Locator Forms (PLFs)

Between 8 June 2020 and 18 March 2022, all persons entering or returning to the United Kingdom from any other country by air were required to complete a UK PLF prior to arrival. PLF data included personal identifiable data, arrival date, place of stay in the United Kingdom and countries visited up to 10 days preceding arrival [14].

Daily (up to the previous day) data extracts were received via transfer using a secure network from the Home Office as per a formal agreement especially formed for the COVID‐19 pandemic response, available from April 2021. Personal identifiers were used to link to COVID‐19 case information in UKHSA's SGSS database through a multistage probabilistic algorithm.

Multistage linkage based on personal information between PLFs and the SGSS cases and variants dataset was carried out to determine and validate a match.

#### 2.1.2 Dataset 2 (Enhanced): Contact Tracing and Advisory Service (CTAS)

CTAS was established as part of the COVID‐19 pandemic response. Contact tracing efforts led by NHS Test and Trace (T&T) collected extensive information from COVID‐19 cases, including whether and where they had travelled internationally preceding onset of symptoms [15]. This included information on passengers travelling by all means of transport (air, rail and sea).

Between December 2020 and 24 February 2022, travel‐associated data were extracted daily (up to the previous day) via a secure network from the CTAS datasets. Similar to Dataset 1, multistage linkage was also carried out with the SGSS cases and variants dataset from June 2021.

#### 2.1.3 Dataset 3: SGSS Travel Datasets

Laboratory test request forms were modified to capture travel information from certain travel‐related testing initiatives, including directed testing of individuals with recent international travel and those in managed quarantine facilities [16, 17]. Travel information included travel status and country of travel within the 14 days preceding the specimen date. These were also extracted on a daily basis.

#### 2.1.4 Dataset 4: Health Protection Surveys

UKHSA Health Protection Teams (HPTs) are an integral part of routine public health surveillance and case investigation. Existing relationships with HPTs were leveraged to supplement gaps in travel information for cases with identified variants of concern.

Between December 2020 and 16 July 2021, SARS‐CoV‐2 cases with a confirmed WGS variant result (Beta, Gamma or Delta) that did not have travel information available from Datasets 1 to 3 were contacted for interview by local UKHSA HPTs. Interviews assessed whether and where the cases had travelled within the previous 14 days.

### 2.2 Assignment of Travel Status

All four datasets were extracted daily with data up to the previous day, independently linked to the SGSS SARS‐CoV‐2 cases and variants dataset on a daily basis, using several patient identifiable variables for deduplication purposes before being combined to create a person‐level journey. The episode‐level travel status was assigned: traveller, contact of a traveller, sporadic and unknown status for episodes that did not link to any of the four datasets (Table 1).

**TABLE 1:** Assignment of travel statuses to individual cases.

Travel status	Description	Confirmed by data sources
Imported	Case is known to have travelled within 14 days prior to their positive test date.	Datasets 1–4
Secondary	Case is a contact of a traveller.	Datasets 2 and 4
Sporadic	Case is confirmed to have not travelled within 14 days prior to their positive test date.	Datasets 2 and 4
Unknown	Assigned under the following circumstances: Case has refused to fill out a survey from T&T or HPTs or was uncontactable. Case answered unknown when surveyed regarding their travel status Case has elapsed their follow‐up period without a response.	Not applicable

### 2.3 Descriptive Epidemiology of the SuITCases Dataset

The integrated SuITCases dataset allowed for descriptive analyses such as summarising age, sex and country/region of travel‐by‐travel status, all of which will be described in the subsequent results section.

## 3 Results

### 3.1 Epidemiological Results

**FIGURE 2:**
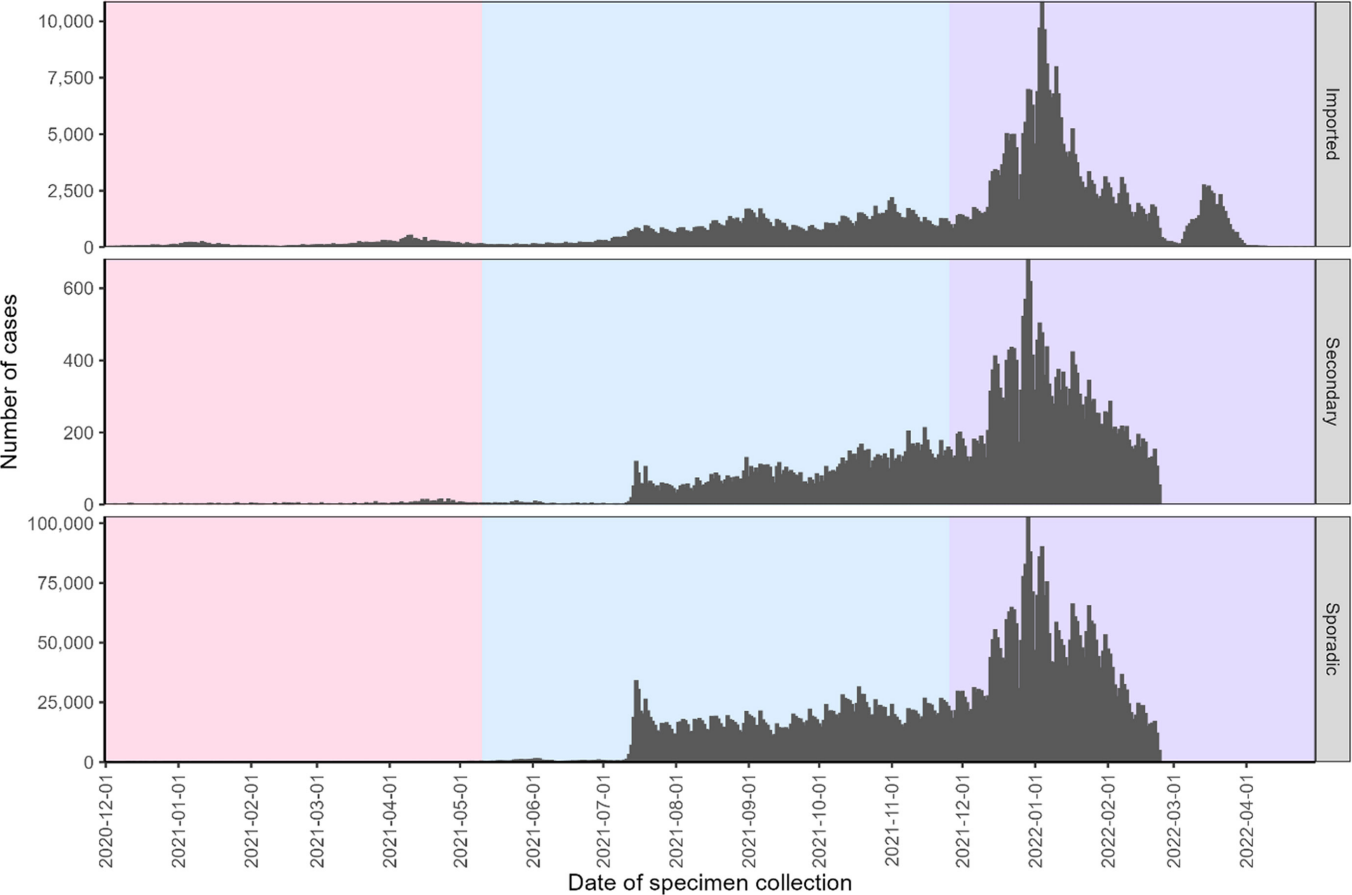
Epidemiological curve of COVID‐19 cases by travel status. *Note:* Excluded 10,093,393 cases where exposure type was unknown. Red shading indicates the SARS‐COV‐2 Beta variant wave (from 18 December 2020), blue shading indicates the SARS‐CoV‐2 Delta variant wave (from 11 May 2021) and purple shading indicates the SARS‐CoV‐2 Omicron variant wave (from 26 November 2021). These dates were when each respective variant was declared as a Variant of Concern by the World Health Organization (WHO).

Between 01 December 2020 and 30 April 2022, 514,988 (3.0% of all national cases) imported, 38,260 (0.2% of all national cases) secondary and 6,467,550 (37.8% of all national cases) sporadic known SARS‐CoV‐2 cases were identified through SuITCases (Figure 2).

**FIGURE 3:**
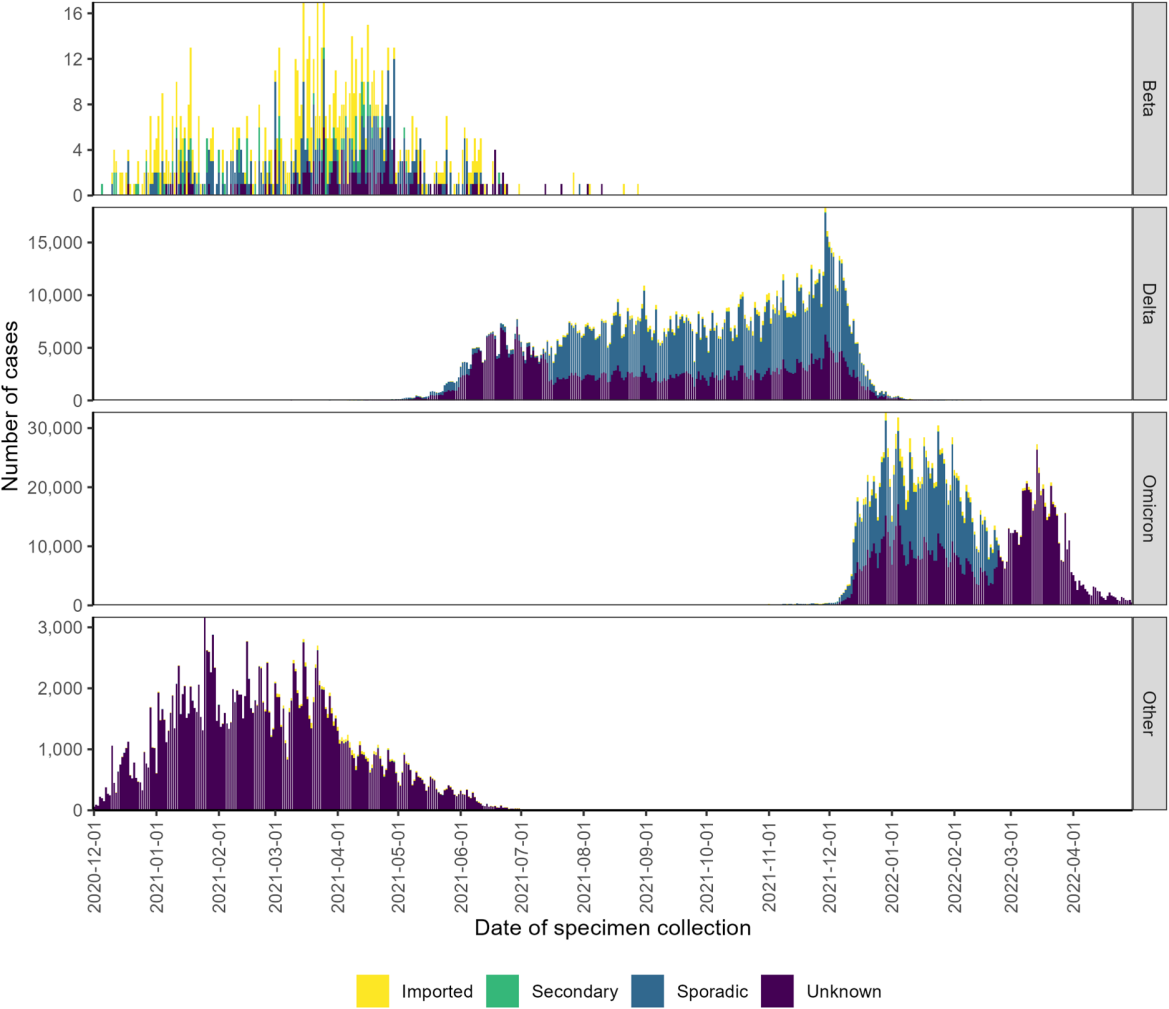
Epidemiological curve of COVID‐19 cases by travel status and variant.

Travel status for SARS‐CoV‐2 Beta, Delta and Omicron variants of concern show that imported cases were most prominent when the Beta variant was first circulating (Figure 3). Although many factors may have influenced the reduction in imported cases over time, the availability of travel information during the Beta wave may have influenced changes in testing strategies and travel bans implemented for subsequent emerging variants.

**FIGURE 4:**
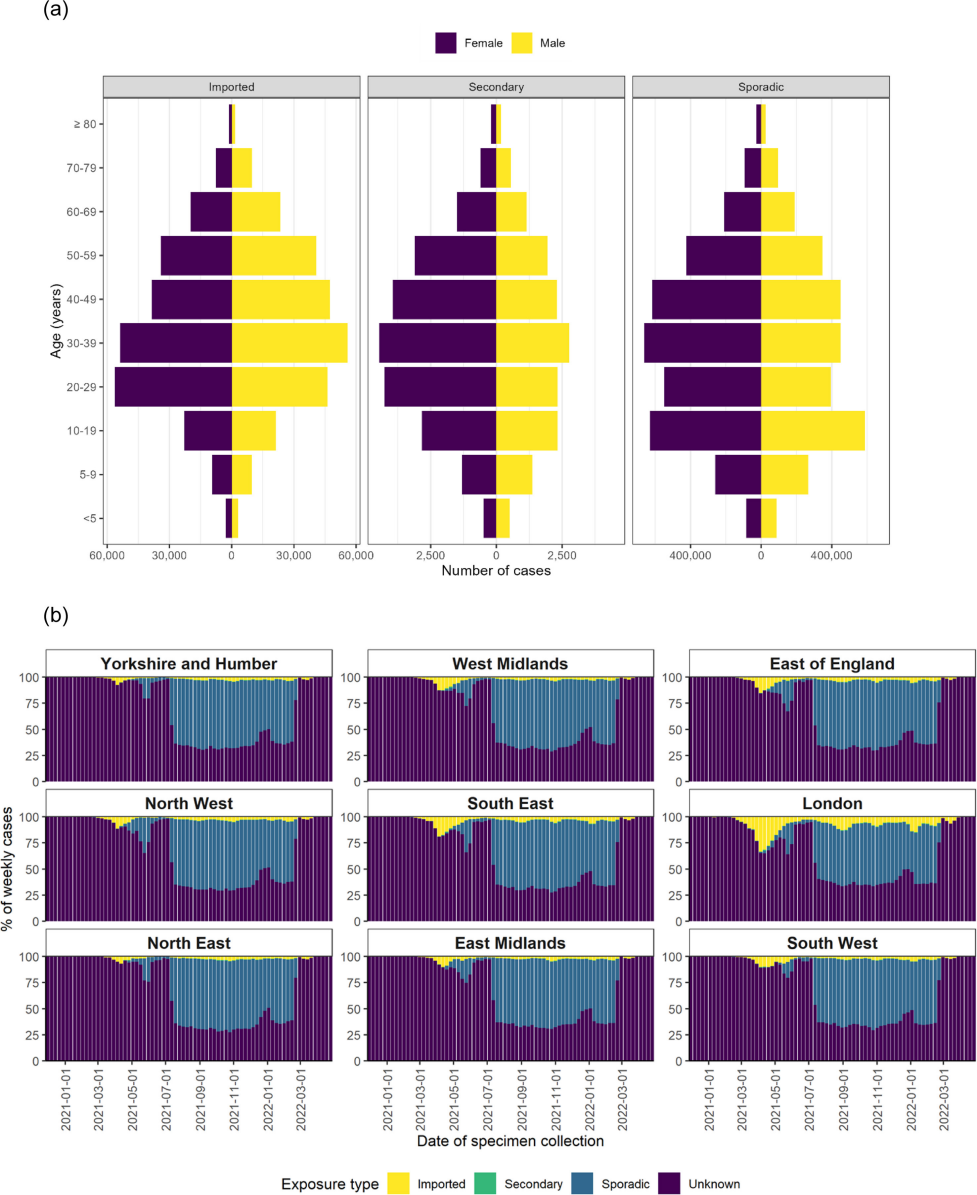
Distribution of COVID‐19 cases by travel status and (a) age–sex and (b) Public Health England Centre (PHEC) regions.

The SuITCases dataset informed public health actions required at the local/regional level by providing distributions of SARS‐CoV‐2 cases by age, sex and region (Figure 4). Imported and secondary SARS‐CoV‐2 cases were mainly in working age groups, and sporadic cases were noted in 10–19‐year‐olds (Figure 4a). The London region experienced a greater proportion of imported SARS‐CoV‐2 cases in early 2021 (Figure 4b).

**FIGURE 5:**
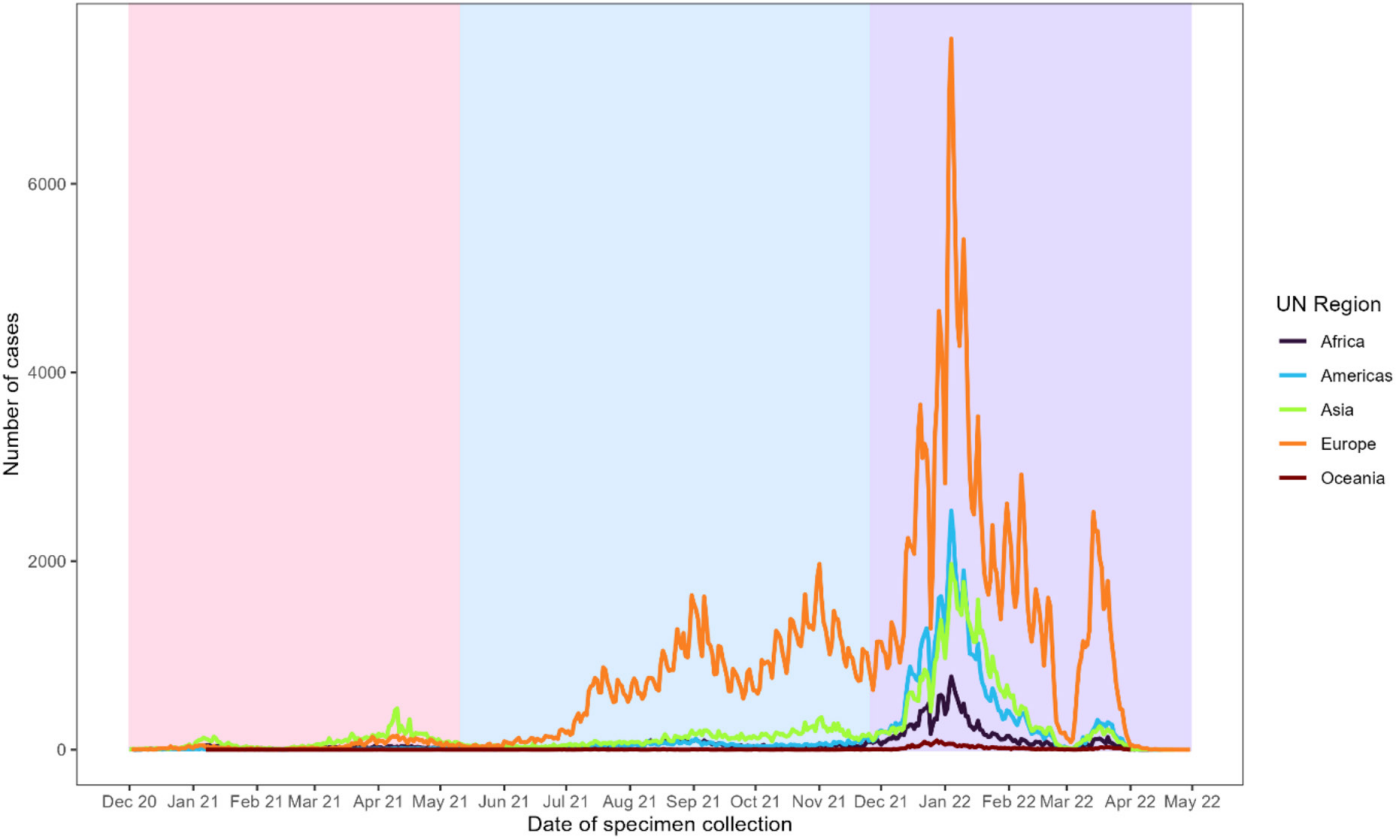
Epidemiological curve of imported and secondary COVID‐19 cases by UN region. *Note:* Red shading indicates the SARS‐COV‐2 Beta variant wave (from 18 December 2020), blue shading indicates the SARS‐CoV‐2 Delta variant wave (from 11 May 2021) and purple shading indicates the SARS‐CoV‐2 Omicron variant wave (from 26 November 2021). These dates were when each respective variant was declared as a Variant of Concern by the World Health Organization (WHO).

The SuITCases dataset was also a source of granular country‐level information to support the national response (Figure 5 and Table 2). This has been highlighted in previous SARS‐CoV‐2 variant‐related studies using this dataset [13, 17, 18]. The epidemiological curve of imported SARS‐CoV‐2 cases by UN region of travel is concurrent with the origin of specific SARS‐CoV‐2 variant waves, such as the SARS‐CoV‐2 Beta and Delta variants emerging from the Africa and Asia regions in early 2021(Figure 5).

**TABLE 2:** Top 10 countries visited by travel‐associated COVID‐19 cases.

Country of travel	Count of associated COVID‐19 cases
Spain	99,275
France	49,408
United States of America	40,948
Italy	24,922
United Arab Emirates	21,186
Portugal	20,812
Poland	18,795
Switzerland	17,179
India	16,236
Greece	15,673

### 3.2 Contribution of Each Data Source to the Integrated System

**FIGURE 6:**
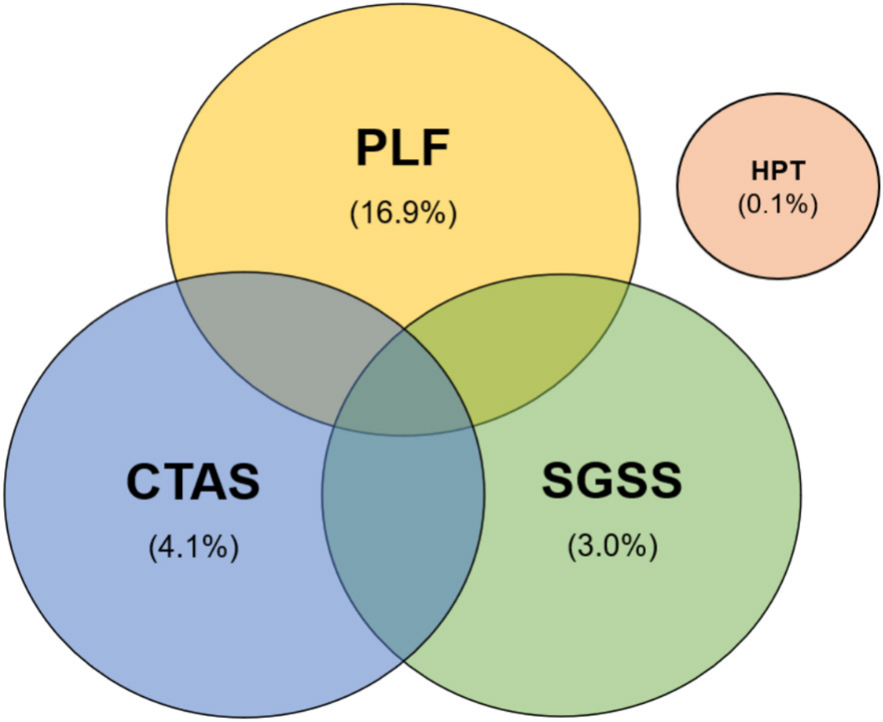
Venn diagram of SuITCases' data sources.

We assessed the proportions of travel‐associated COVID‐19 cases by each data source to understand relative contributions to the overall integrated SuITCases system. Of the 514,988 travel‐associated SARS‐CoV‐2 cases between 01 December 2020 and 30 April 2022, the large majority were identified from a combination of two or three of the data sources (38.3% and 37.8% respectively) (Figure 6).

PLFs were the largest individual data source and contributed to assigning 39.8% of travel‐associated cases, followed by 27.6% through CTAS, which were the two enhanced systems established during the COVID‐19 pandemic.

## 4 Conclusion

SuITCases played a vital role in informing the COVID‐19 pandemic response in England by identifying travel‐associated SARS‐CoV‐2 cases/variant cases, including both imported and secondary cases. This not only informed rapid public health action like travel‐related public health and social measures and increased surge testing but also contributed to wider epidemiological studies and assessments such as assessing the risk of hospitalisations due to SARS‐CoV‐2 variants [13, 17, 18, 19].

In conclusion, this methods study highlights the successful implementation of a surveillance system, SuITCases, to robustly capture travel‐associated SARS‐CoV‐2 cases by integrating multiple data sources to inform rapid public health actions. The benefits of leveraging enhanced data collection (PLFs and CTAS) in combination with routine surveillance systems should be considered as potential tools for future enhanced investigations and pandemic preparedness plans for emerging infections.

## Author Contributions

**Mary A. Sinnathamby:** conceptualization, methodology, writing – original draft, formal analysis. **Katherine Twohig:** methodology, data curation, formal analysis, writing – review and editing, conceptualization, visualization. **Nurin Abdul Aziz:** methodology, data curation, formal analysis, writing – review and editing, visualization. **Florence Halford:** writing – review and editing. **Asad Zaidi:** conceptualization, methodology, data curation, writing – review and editing, visualization, formal analysis. **Katie Harman:** visualization, writing – review and editing, methodology. **Simon Thelwall:** supervision, writing – review and editing. **Alex Allen:** writing – review and editing, supervision. **Gavin Dabrera:** conceptualization, methodology, writing – review and editing, supervision.

## Conflicts of Interest

The authors declare no conflicts of interest.

## Peer Review

The peer review history for this article is available at https://www.webofscience.com/api/gateway/wos/peer‐review/10.1111/irv.70141.

## Data Availability

Data requests should be submitted to the UKHSA Office for Data Release, available from: Accessing UKHSA protected data ‐ GOV.UK (www.gov.uk).
